# Non‐Facial Skin Rejuvenation of the Neck, Chest, and Hands. Part One: Using Injections

**DOI:** 10.1111/jocd.16624

**Published:** 2024-11-15

**Authors:** Mariana El Hawa, William Abou Shahla, Callie Fares, Dana Saade

**Affiliations:** ^1^ Department of Dermatology American University of Beirut Medical Center Beirut Lebanon

**Keywords:** fillers, injections, lasers, non‐facial, rejuvenation, skin

## Abstract

**Background:**

The demand for aesthetic procedures aimed at restoring and preserving a youthful appearance is growing. While numerous non‐surgical facial rejuvenation techniques are available, there is a need for a comprehensive review of clinic‐based procedures targeting non‐facial body parts.

**Aims:**

This review aims to describe and evaluate clinic‐based techniques for rejuvenating the neck, chest, and hands, focusing on various types of fillers and other non‐invasive procedures.

**Methods:**

In this first part of this review, we conducted an extensive literature review on PubMed, reporting the effectiveness of different fillers, detailing their preparation, required volume per area, injection methods, durability, and any associated side effects. We also discuss the use of mesotherapy, microneedling, chemical peeling, and Profhilo in these specific body areas.

**Conclusion:**

The review synthesizes the latest data on the effectiveness and safety of these procedures, highlighting the advancements in filler technology and the growing popularity of non‐invasive techniques for body rejuvenation. This article serves as a valuable resource for practitioners and patients interested in non‐surgical body rejuvenation, providing insights into the best practices.

## 1 Introduction

Skin rejuvenation is defined as a cosmetic treatment aiming at restoring a more youthful appearance [1]. While techniques for rejuvenation of the face, such as facelifts and dermal fillers, have been practiced for several decades, there has been a lack of focus on rejuvenating other body parts. Restricting rejuvenation efforts to the face alone creates a noticeable contrast with the aging of the neck, chest, and hands. In recent times, new techniques have gained popularity, shifting the focus toward rejuvenating the skin of non‐facial body parts for a more overall youthful look [2, 3]. Our review aims to describe clinic‐based techniques available to rejuvenate non‐facial body parts, specifically focusing on the hands, neck, and chest.

The entirety of the neck is covered by the platysma muscle, which stretches from the skin of the lower lip down laterally and medially and passes the clavicle [2]. Age can be due to intrinsic causes leading with time to thinning of the platysma muscle giving an aged appearance of the skin [2]. The muscle goes from being diffuse and uniform to a series of fibrous bands [4]. This is reflected with the appearance of horizontal rhytids and sagging skin. With an attachment at the corners of the mouth, the platysma is able to stretch the mouth downward, giving a saddened look. Initially, a thin layer of subcutaneous fat is present between the platysma and skin; however, with time, this layer begins to disappear removing the barrier of the muscle to the skin [4]. Subplatysmal fat is located between the superficial platysma and the deeper strap muscles covering the larynx. For the rejuvenation of this part of the neck, surgical options including procedures such as liposuction, a submental neck lift, a short‐scar face and neck lift, and a full‐scar face and neck lift could be done [5]. Extrinsic aging of the skin is also seen, due to ultraviolet radiation and other external exposures. Contemporary techniques done on the neck for rejuvenation include fillers, botulinum toxin, sclerotherapy, ultrasound techniques, microneedling therapy, thread lifting, radiofrequency therapy, lasers, chemical peels, and light sources.

Causes of aging in the chest are like that of the neck, due to both intrinsic features and extrinsic exposure. The chest has dermal and epidermal layers that are thinner than the face, making it more prone to aging due to the reduced number of pilosebaceous glands [6]. Moreover, in some patients, platysma fibers extend beyond the second and third ribs, making chest rhytids more apparent. The origin of the platysma muscle is from the clavicle, acromial region, pectoralis muscle, and the deltoid muscles. Similar to the neck, the muscle becomes less well defined and thinner [7]. These factors make certain techniques difficult to perform on the chest, as will be discussed further in this review. Most often, a combination of therapy may be needed for a severely photodamaged chest. The goal of treating the chest is to create a uniform appearance from the face down, making the treatment of the chest an essential step in the rejuvenation journey [8]. The current trends for the rejuvenation of the chest are similar to that of the neck.

The hands are the second most exposed part of our body to environmental stresses, after the head. The thin skin of the dorsum of the hand is subject to various morphological changes with aging. Extrinsic and intrinsic factors are involved in the process of aging hands. Essentially, extrinsic factors such as UV light, environmental chemicals, and smoking lead to apparent changes at the level of both the dermis and epidermis manifested as solar lentigines, premalignant actinic keratosis, and hypopigmentation. On the other hand, the damaged microcirculation causes fat loss and dermal atrophy leading to the subsequent appearance of rhytids and the prominence of veins, bones, and tendons. Studies have shown that aging hands can reveal the true age of the person, especially if contrasted with a rejuvenated face and neck [9]. Multiple studies have produced five‐point scales to assess for the severity of volume loss as a universal grading system. These scales include the degree of fatty tissue loss, the visibility of tendons and veins, and the texture of the skin, along with wrinkling and pigmentation [10, 11]. Current methods to rejuvenate the hands include volume restoration with fillers, sclerotherapy, superficial chemical peels, and lasers. Most often a combination of these treatments is needed to achieve the desired result.

*This is the first part of a two‐part series focused on the non‐facial rejuvenation using fillers and skin boosters.*

## 2 Fillers and Skin Boosters

Fillers are injectables defined as implantable medical devices by the US Food and Drug Administration (FDA). Fillers include numerous products that differ in their mechanisms of action, chemical composition, duration, safety and side effects, and interaction with the host tissues. Fillers can be injected either through a needle or a cannula and the depth of injection varies according to the site of injection, the indication, and the desired endpoint.

Under the category of fillers, there exist skin boosters that have gained popularity in the recent decade. While dermal fillers are usually injected into the deep skin layers including the deep dermis and subcutaneous fat to restore volume, skin boosters are usually injected into the dermis to hydrate and revitalize the skin. Skin boosters are the perfect treatment for patients who are looking to have smoother and healthier skin. Several types exist in the market which differ in their composition and method of use.

In the following section, we will tackle the three most used dermal fillers: poly‐l‐lactic acid (PLLA), calcium hydroxyapatite (CaHA), and hyaluronic acid (HA).

### 2.1 Poly‐l‐Lactic Acid (PLLA)

PLLA was initially approved in 2004 by the FDA for use in HIV lipoatrophy, and later in 2009 gained approval for use in nasolabial folds, contour deficiencies, and facial wrinkles [12]. It is now used off label for the rejuvenation of the neck [2], chest, hands, and thighs (Table 1). Commercial brand names of PLLA are Sculptra (Dermik Laboratories, Sanofi‐Aventis, Bridgewater, NJ, USA), DevoluxTM PLLA (Dermax Co, China), or GANA FILL (Fit Peptide Co., Hong Kong, China). PLLA is a biocompatible, biodegradable injectable filler that produces volume restoration through fibroplasia and the formation of new collagen. Thus, in addition to increasing volume in that area, it improves skin texture and tightness [13]. Finally, when PLLA is injected, it causes a “tissue‐based inflammatory reaction,” which means that each patient will react differently.

**TABLE 1:** PLLA injection techniques, adverse events, and post‐procedure care on the neck, chest, and hands.

Filler type	FDA approved	Preparation	Volume needed	Injection sites	Method of injection	Durability	Follow‐up session	Side effects	Instructions after the injection
PLLA									
Neck	No—off label [2]	1 vial diluted in 8–10 mL water with 0.1 mL of 2% lidocaine kept undisturbed for 3–4 h [13, 14]	12 mL, 0.5 mL per injection [13]	Demarcate the injection sites by squares separated by 1 cm each over the entire neck area	Punch the skin between thumb and index. Point‐by‐point injection using an 18G needle [13]	Up to 2 years or longer [13]	Wait 4–6 weeks until the second session, and then another 8–10 months if a third session is needed [2]	Ecchymosis, edema, pain, pruritus, inflammation, and hematomas [15] Appearance of subcutaneous nodules [13, 14] Foreign body granuloma [16]	Massage the area for 2–5 min, 3 times a day for 7 days [13]
Chest	No—off label [15]	1 vial with 6–10 mL sterile water with 2 mL of 1% lidocaine then agitated using a mixing device [13] or Withdrawing 1.5 mL of PLLA and lidocaine and mixing it with 1.5 mL water until reaching 16 mL [3] Or 1 vial with 1 mL of 1% lidocaine	16 mL, 0.05–0.1 mL per injection [15]	In the center of the rhytids located between the breasts and then laterally and superiorly following the subcutaneous fat plane, utilizing a retrograde linear threading technique [17]	Using a 27G needle, fanning technique [3] or point‐by‐point [13]	Up to 2 years or longer [18]	Monthly for 3–4 months	Erythema and bruising, infection and subcutaneous nodules [3]	Massage the area for 5 min, 3 times a day for 7 days [13]
Hands	No—off label [19]	1 vial in 5–12 mL of water and 1 mL of 1% lidocaine [20, 21]	2–5 mL, 0.3–0.5 mL per injection	Dorsal hand at the midpoint length of the hand [20] Intermetacarpal space 0.3–0.5 mL [20]	Using a 25–27G needle, linear threading technique with a retrograde injection [20]	2 years or longer	Between 2 and 6 follow‐up sessions are needed, at the 4 weeks' interval for the first few sessions, then possibly at 3‐month interval	Bruising, edema, itching, subcutaneous nodules, etc. In addition, arterial spasm can rarely occur, which resolves with a five‐minute massage [20]	Massage the injected areas three times daily for 1 min for 3 days [19]

#### 2.1.1 Neck

At the level of the neck, the choice of filler is critical due to the risk of nodules and beading along injection sites; this can be resolved but preferably avoided [22]. A small volume of local anesthetic such as lidocaine is added to the vial and swirled gently to allow even suspension. The solution is injected at an angle of 60° with the bevel facing down [13, 14], reaching the transition between deep dermis and subcutaneous tissue [13]. The current recommended injection technique is point‐by‐point (Table 1).

Results appear earlier in the chest and neck than in the face; where they are seen in 10 to 30 days versus over a month, respectively [13]. The effects of PLLA last much longer compared to other fillers, with reported duration of up to 2 years or more [13]. A major complication, mainly a product‐related adverse event, is the appearance of the subcutaneous nodules. It is important to educate the patient about the frequency of those events to set expectations prior to the injection. To avoid them, massaging the areas treated after the procedure is crucial [13, 14]. If they do appear, palpable nodules can be treated with an injection of sterile water and massage or, in severe cases, with intralesional triamcinolone [14]. Other useful tips are avoiding intradermal injections and excessive quantities of product, and spacing treatment sessions no sooner than 4 weeks apart. Another rare complication is the formation of foreign body granulomas due to hypersensitivity reaction to PLLA. The frequency of these side effects can be reduced if the patients are instructed to avoid anticoagulant or antiplatelet medications prior to the procedure, as well as vitamin E and fish oil supplements [16].

#### 2.1.2 Chest

At the level of the chest, PLLA needs a specific preparation. PLLA is not FDA approved for the chest's rejuvenation [19]. Following the necessary preparations, injections start within the rhytids between the breasts, proceeding laterally and superiorly along the subcutaneous fat plane using a retrograde linear threading technique. The treatment area extends superiorly at the suprasternal notch, laterally to the midclavicular line, and to the fourth rib inferolateral [17]. The advantages of the point‐by‐point technique are greater control over the amount injected and less risk of pain and hematomas [13]. In the chest, the post‐procedure massage is as important, as in other body areas, to avoid the formation of nodules and we can educate the patients to perform proper massaging. Treatment should be repeated monthly for 3–4 months to get the best results, and the correction is expected to last up to 2 years and beyond [18]. Like any other filler injection, expected side effects can be injection site reactions as described previously [3].

#### 2.1.3 Hands

For the hands, 1 vial of 150 mg of PLLA is diluted in 5–12 mL of water [20, 21]. Local anesthetic can be added to the solution, around 0.5 mL of Mepivacaine or 1% Lidocaine [20]. Prior to the injection, a topical anesthetic can be applied, and the injection sites should be cleansed. It is important to stop injecting before the needle exits the skin to avoid superficial deposition of the filler. At the level of the intermetacarpal space, the skin is tented in between the tendons and a small amount of filler, and 0.3–0.5 mL of solution is injected [20]. The total amount of filler injected is 2–5 mL per hand (Table 1). The injected area should be massaged during the procedure to distribute the filler evenly in the hand. Patients are also instructed to massage the injected areas three times daily for 1 min for 3 days [19]. More diluted solutions can be used for the touch‐ups [20, 21]. Expected side effects are like those of the neck. In addition, arterial spasm can rarely occur, which resolves with a five‐minute massage [20].

### 2.2 Calcium Hydroxyapatite

CAHA is a biodegradable, biocompatible, and resorbable bio‐stimulator filler substance consisting of hydroxyapatite microspheres in a 70% carboxymethyl cellulose carrier gel [23, 24]. CaHA received FDA approval in 2006 for facial soft tissue augmentation including the correction of moderate to severe nasolabial folds, and the restoration and correction of the signs of HIV‐associated facial lipodystrophy. Brand names include Radiesse (Merz Pharmaceutical), Renu (Medyglobal), and Facetem (Daewoong Pharmaceutical). Its unique feature is that it contributes to volumization, immediate correction, and collagen biostimulation, gradual correction. The deposition of the filler allows gradual tissue formation by causing neo‐callogenesis, elastin deposition, angiogenesis, and dermal cell proliferation [25]. Its composition causes negligible immunogenicity as it has a similar composition as human bone and teeth [23].

Although not typically used as a first‐line treatment, CaHA has been used for the rejuvenation of the neck, both alone and in combination with other techniques (Table 2) [36]. Numerous injection techniques and dilutions are proven to be efficacious. In 2009, the Food and Drug Administration granted approval for a protocol that involves blending CaHA with lidocaine at a 0.3% concentration to enhance patient comfort during the injection process. It is injected with a 0.5 in. 27‐G needle using the linear threading technique. Moreover, the majority of experts (90%) agreed on the fanning and retro‐injection technique via cannula with 3 to 5 entrance points in a study aiming at conducting a consensus recommendation for the use of CaHA [25]. Effects of CaHA usually last 15–18 months. Expected side effects include early formation of nodules [26], persistent inflammation, swelling, or erythema [27]. Late side effects include granulomatous foreign bodies, migration of filler material, and persisting skin discoloration [27].

**TABLE 2:** CAHA injection techniques, adverse events, and post‐procedure care on the neck, chest, and hands.

Filler type	FDA approved	Preparation	Volume needed	Injection sites	Method of injection	Durability	Follow‐up session	Side effects
CAHA								
Neck	Off label	Mixing 1.5 mL of CaHA with 0.5 mL of 2% lidocaine solution and 2.5 mL of 0.9% saline solution Another diluted method for the vertical lines: A volume of 1.3‐mL of CaHA mixed with 1.4 mL of 2% lidocaine (1:1 dilution) [25]	4.5 mL	3–5 entry points, 2 on each lateral side of the neck and around the submaxillary region [25]	Using a 22‐G needle using the fanning technique or 25G and a 50 mm long cannula	15–18 months	Not clear if a touch‐up session is necessary after the first session	Early side effects: formation of nodules [26] persistent inflammation, swelling, or erythema [27] Late side effects: include granulomatous foreign bodies, migration of filler material, persisting skin discoloration [27]
Chest	Off label [28, 29]	CaHA can be diluted in 3–6 mL of diluent, usually sterile water, with 1:2 or 1:4 dilution [25]	0.5–1 syringes of the total volume, injected in the décolletage [25]	Injected in the décolletage	Two most described methods of injection: short linear threading technique using a needle or retro‐injections using the cannula (fanning or asterisk)	15–18 months	Not clear if a touch‐up session is necessary after the first session	Same side effects as one from other body areas
Hands	Yes Since 2015	0.75–1.3 mL of Radiesse with 1% or 2% lidocaine solution in a 1:1 ratio [30]	1–2.25 cc, per hand [31]	3–5 insertion sites of the dorsal hand, until a nodule is raised [30]	The skin is tented up, then using a 25‐G 1.5 in. needle Or serial puncture with a needle, fanning technique using a cannula or microdroplets technique with a needle [32]	Between 12 to 15 months [33, 34]	One session is usually enough without any touch‐up sessions [30] or can reach 2 sessions [32]	Causes pain, bruising, erythema, edema, and itching [33] Minimal risk of foreign body reactions [34, 35]

#### 2.2.1 Chest

There exist few data for the use of CaHA in the décolletage area, as it is not typically used for the rejuvenation of this body area. CaHA can be diluted in 3–6 mL of diluent, usually sterile water, with 1:2 or 1:4 dilutions. The two most described methods of injection are short linear threading technique using a needle or retro‐injections using the cannula (fanning or asterisk).

#### 2.2.2 Hands

CAHA has been FDA approved since 2015 for the rejuvenation of the hands. A volume of 0.75–1.3 mL of Radiesse with 1% or 2% lidocaine solution in a 1:1 ratio is first put together [30, 31]. The skin is tented up, and then using a 25‐G 1.5 in. needle, the volume is injected at three to five insertion sites of the dorsal hand, until a nodule is raised [30]. The injection techniques that can be used are serial puncture with a needle, fanning technique using a cannula or microdroplets technique with a needle [32, 37]. The proximal‐to‐distal fanning technique using a cannula was preferable compared to other techniques in one study while the fanning and the multipoint technique were comparable in another [38].

A volume between 1 and 2.25 cc, per hand produces desirable outcomes. The higher the dilution, the more the injection can be made superficially, with less product visibility and nodule formation [31]. In general, CaHA, like any other filler, causes pain, bruising, erythema, edema, and itching [33]. It has minimal risk of foreign body reactions [34, 35]. One case report reported acute exacerbation of carpal tunnel syndrome as an adverse event of CAHA injection into the hands [39].

### 2.3 Hyaluronic Acid

HA has been used as a dermal filler since 2003. It is a natural glycosaminoglycan composed of glucuronic acid and N‐acetyl glucosamine disaccharide units [23]. Currently, several HA brand names are approved in the market, where the products differ in their substance composition. Manufactured HA filler can be cross‐linked or not. Adding cross‐linking agents contributes to more density and durability of the product [34]. HA is considered an inert substance, as it is ubiquitous in the extracellular matrix of humans, and thus its immunogenicity is minimal [40]. The hyaluronidase family of enzymes can be used to catalyze HA and reverse its effects when needed.

Most skinboosters are composed of nonanimal stabilized HA (NASHA). Examples are Restylane Vital (Galderma, Uppsala, Sweden), composed of 20 mg/mL of partially cross‐linked small particle HA, Teosyal redensity 1 composed of 15 mg/mL non‐cross‐linked HA in addition to other multiple vitamins and proteins, and Juvederm Volite which is composed of 13 mg/mL cross‐linked HA.

#### 2.3.1 Neck

At the neck, a topical anesthetic is applied and sterilization of the skin is performed. Products like Restylane Lyft are already mixed with lidocaine and water, and therefore no dilution is necessary. If non‐mixed products are used, 1 mL of HA is prepared with 0.1 mL of 2% xylocaine. A needle is generally preferred over a cannula [41]. The length of the effect is 6 to 8 months [15]. Side effects that one may expect are edema in 25% of the patients [42], redness, pain, and blue discoloration which may resolve after 7 to10 days [41]. Other adverse events like extremity pain and pruritus can also occur [43, 44, 45]. Rare side effects are the formation of granulomatous nodules [46, 47] in addition to fungal and bacterial infections [47, 48].

As mentioned before, skin boosters are injectable products that are slightly different from dermal fillers which can also be injected at the level of the neck. While most skin boosters are composed of HA, they contain small particles of HA which help in delaying aging. They revitalize the skin at the level of the neck by increasing hydration to the treatment area. In the neck, it is usually injected by means of intradermal micropuncture with small aliquots of product on the horizontal lines 4 cm away from the midline (Table 3) [53].

**TABLE 3:** HA injection techniques, adverse events, and post‐procedure care on the neck, chest, and hands.

Filler type	FDA approved	Preparation	Volume needed	Injection sites	Method of injection	Durability	Follow‐up session	Side effects	Instructions after the injection
HA									
Neck	Off label [49]	Products like Restylane are already mixed so no dilution needed If non‐mixed products, 1 mL of HA prepared with 0.1 mL of 2% xylocaine	1 mL per side and 2 mL overall is usually enough [41]	The product is then injected parallel to the skin using the retrograde linear technique [17, 42]	Needle is preferred over a canula [41] 30G sharp needle for finer, more superficial contours 25‐G blunt cannula for wider, deeper contours	Results can be noted after a month [42] The length of the effect is 6–8 months [15]	Touch‐up can be done 1 month later	Edema, redness, pain, and blue discoloration which may resolve after 7–10 days [41] Others like extremity pain and pruritus can also occur [43, 44, 45] Rare side effects are formation of granulomatous nodules [46, 47] in addition to fungal and bacterial infections [47, 48]	
Chest	Off label	Either a Restylane Vital Light (Europe) or Restylane Silk +0.5 mL of Lidocaine 10% without epinephrine (USA) Or or 2–3 mL of the NASHA diluted in saline in 1:4 [17]	About 0.02 mL is released at each injection point, using a total of approximately 2 mL	1–1.5 cm apart	4 mm needle is inserted at an inclination of 45° intradermally, using the serial puncture technique or the retrograde linear threading technique [17]	Can last for up to 6–8 months [3]		Mild bruising and redness [6]	
Hands	Yes For volume restoration (Walker K)	The preferred dilution is 1:1 with 0.8 mL saline and 0.2 mL of 1% lidocaine with small amounts of epinephrine [34]	0.5 mL per web space and 1–4 mL per hand [50]	Injected in a perpendicular way in the superficial and intermediate dorsal layers, avoiding the deep layers The threading serial puncture, small bolus, and fanning techniques were all reported injection techniques	The use of cannula was associated with more discomfort as compared to needle [43]	Results usually last about 6–12 months [32]	Only one session is usually enough unless the first session gave sub‐optimal results [44, 45, 50, 51]	Similar side effects can be expected t in the hands with HA as other fillers in other body areas	Massaging the area injected has been proved to be essential in allowing the filler to sit in the desired structures Other experts may also advise to sit on the involved hand immediately after the procedure [52]

#### 2.3.2 Chest

At the level of the chest, the treatment area depends on the patient's preference and choice of clothes. Inject 2–3 mL of nonanimal, stabilized hyaluronic acid (NASHA) diluted with saline in a 1:4 ratio (1 mL of NASHA to 3 mL of bacteriostatic normal saline) [17]. However, one can also use manufactured syringes containing lidocaine and water like Restylane Lyft and Belotero Balance Lido, injected in the superficial dermis by applying a retrograde linear threading technique [54]. Improvements in wrinkle reduction were seen up to 120 days. No pain was reported during or after the injection, but mild bruising and redness were seen [6]. Other side effects are similar to other body areas. Some reports note that results can last in the chest for up to 6 to 8 months [3].

HA in the form of Skinboosters can also be injected into the décolletage and chest. It is indicated when the skin loses both its elasticity and quality. Injections are done in each of the three edge points of the V‐neck in a retrograde fashion using a blunt cannula or direct injections [36]. Around 1–2 mL is injected into the treated area.

#### 2.3.3 Hands

HA, more specifically, Restylane Lyft is approved for facial folds and wrinkles, cheek augmentation, and dorsal hand volume restoration by the FDA [55]. Manufactured syringes can be used directly in the form of either Restylane Vital Light as found in Europe or Restylane Silk as found in the USA, which consist of the HA and 0.3% lidocaine to reduce pain during injection [56, 57]. When preparing non‐diluted products, the preferred dilution is 1:1 with 0.8 mL saline and 0.2 mL of 1% lidocaine with small amounts of epinephrine [34]. After application of topical anesthesia, HA filler is injected in a perpendicular way in the superficial and intermediate dorsal layers, avoiding the deep layers. The use of cannula was associated with more discomfort as compared to the needle [43]. Most studies found satisfactory responses when using 0.5 mL per web space and total of 1–4 mL per hand [44, 50, 52]. Massaging the area injected has been proven to be essential in allowing the filler to sit in the desired structures. Other experts may also advise sitting on the involved hand immediately after the procedure [52]. Results usually last about 6 to 12 months [32]. Similar side effects can be expected in the hands with HA as in other body areas.

HA as a skinbooster is also used on the hands. A volume ranging from 0.4 to 1 mL is usually needed [52, 53, 58]. Manual techniques described in the literature consist of threading [52] or tunneling techniques [58]. It is important to inject distal to the wrist and massage the hand downward using an anti‐bruising cream and melting ice [52]. Touch‐ups are usually needed, but the number depends on the type of skinbooster, which is usually three sessions spaced 1 month apart [53, 59].

## 3 Mesotherapy

Initially described in France by Dr. Michel Pistor, intradermal therapy or mesotherapy is a non‐surgical procedure that aims at injecting substances in the superficial dermis like drugs in order to treat various skin conditions [60, 61]. In aesthetic medicine, injection of various vitamins, proteins, and minerals leads to skin rejuvenation. This easy‐to‐perform procedure induces the biosynthesis of fibroblasts, enhancing cell activity and synthesis of collagen, elastin, and HA. The end result is glowing, tight, and moisturized skin. HA, already discussed as a dermal filler and skin booster, is often preferred for mesotherapy due to its ability to increase hydration. Another type of mesotherapy called injection lipolysis—which will not be discussed in this review—is aimed at contouring by means of dissolving excess fat using agents like phosphatidylcholine (PC) and deoxycholic acid (DC) [62].

There is usually no downtime after the procedure. As for the side effects, minor post‐injection events such as erythema, ecchymosis, and edema can occur. Infection caused most by atypical mycobacterium can occur months after mesotherapy if aseptic precautions were not taken appropriately (Table 4) [65, 66].

**TABLE 4:** Mesotherapy injection techniques, adverse events, and post‐procedure care on the neck, chest, and hands.

	FDA approved	Preparation	Volume needed	Injection sites	Method of injection	Follow‐up session	Side effects
Mesotherapy							
Neck and chest	No Vitamins and minerals are not considered drugs	32 mg of HA mixed with amino acids, nucleotides, antioxidants, and vitamins	5 mL	Injected into the superficial dermis	Microinjection technique and 23 G needle	Four sessions separated by 3 weeks	Minor post‐injection events such as erythema, ecchymosis/edema/infection can occur (Van dissel)
Hands	No Same reason	Solution containing HA, vitamin A, vitamin C and E	1 mL solution	Injected into the superficial dermis at different points spaced 2 mm apart The depth of injection does not usually exceed 2–2.5 mm [63]	The picotage method inclined at 45°, a 30 G needle Or a meso‐gun can also be used	One session is done every week for 4 weeks and then one session every month for 3 months [64]	

### 3.1 Neck and Chest

Sparavigna et al. studied the effect of a solution composed of 32 mg of HA mixed with amino acids, nucleotides, antioxidants, and vitamins on the face and other body areas of 64 patients. Results showed a reduction in the senile lentigines, improved hydration and brightness, and rejuvenation of the skin with at least first‐grade improvement in the severity as per the severity rating scale [64].

### 3.2 Hands

At the level of the hand, a mesotherapy product is best injected using the picotage method. Inclined at 45°, a 30 G needle is injected into the superficial dermis at different points spaced 2 mm apart. Note that a meso‐gun can also be used. The depth of injection does not usually exceed 2–2.5 mm [63]. In one study, a volume of 1 mL solution containing HA, vitamin A, vitamin C, and E was injected using the Microinjection technique on 20 patients. One session was done every week for 4 weeks and then one session every month for 3 months. Out of all the treated patients, 16 pairs of hands were improved in terms of glow and elasticity according to an evaluation scale [67].

## 4 Microneedling

Microneedling or percutaneous collagen induction is a minimally invasive procedure that uses sharp needles to induce direct mechanical trauma to the skin. The regenerative effect of this technique stems from the fact that the induced micro‐wounds will stimulate the scarless wound healing cascade by means of inflammation and remodeling. With minimal epidermal damage, the disruption of tissues leads to fibroblast deposition of collagen (types III and I), neovascularization, and neocollagenesis thus elevating and filling in the furrows caused by aging. This process may take weeks and months, and thus improvement in the skin continues to be noticeable after a session of microneedling [62, 68]. In addition to rejuvenation, microneedling has been used to treat post‐acne or burn scars, melasma, enlarged pores, and androgenetic alopecia and alopecia areata [68]. There exist several types of instruments and needles. In general, the FDA classifies microneedling devices as class II with a moderate risk level. It is authorized for use for acne scars, facial wrinkles, and abdominal scars. Microneedling in other body areas is still not approved. The first type of device is a rolling device, or a dermaroller, which is a drum‐shaped tool with a cylindrical head that is applied on the skin back and forth to induce micropores in the papillary dermis [69]. It comes in different shapes and sizes depending on the brand, and some devices can be utilized for home use. Other clinic‐based microneedling devices consist of electronic pens. While it is not possible to manipulate the penetration depth of the needle with a dermaroller, electronic pens such as MDPen or SkinPen have modifiable speed and depth which makes them advantageous. Microneedling pens can also be used for the delivery of radiofrequency or products like hyaluronic acid or vitamins or platelet‐rich plasma. Fractional microneedling radiofrequency FMRF device uses thermal injury by the generation of electric current through insulated microneedles [69]. Another innovative type of device is the light‐emitting microneedling device which includes titanium microneedles and LED light.

Patients who are advised not to undergo microneedling are those who have allergies to anesthetics or other skin care products, poor wound healing and keloidal tendencies, severe acne, elastosis, eczema or psoriasis, or are on anticoagulants [70]. Optimally the skin should be prepared with vitamins A and C 1 month prior to microneedling for maximal results [71]. A downtime of 2–3 days usually occurs after a session; patients may experience mild erythema, edema, and skin flaking. Sunscreen should be used, and sun exposure should be avoided after the session. HA products and vitamin A products are advisable after a few days.

### 4.1 Neck

Microneedling can be done to improve skin aging at the level of the neck. Out of eight participants in a study conducted by Hou et al., seven benefited from only two sessions of microneedling [72]. After disinfecting the neck and applying a topical anesthetic, they used Dermaroller MF8 (LLC, Thousand Oaks, CA) composed of 192 needles and nine rows. The needles with a length of 1.5 mm and a diameter of 0.25 mm were rolled over the neck in four directions four times: horizontally, vertically, and diagonally right and left [73]. Improvement was evident clinically but also by ultrasound; after 32 weeks, the dermis had an increased thickness with an average of 0.45 mm. Pinpoint bleeding, the treatment endpoint, is usually self‐resolving within minutes of the procedure. Noninsulated radiofrequency microneedling is also proved to be effective when done in the neck area. Xiao et al. used 25 noninsulated gold‐plated microneedles electrodes, to a treatment field with a 300‐μm diameter and 1.8–2.5 mm penetration depth with pulse duration of 80–100 ms at a power of 12–14 W. They found this treatment highly efficacious, and improvement was seen after 1–3 sessions in 50% of the patients after 3 months [74]. In another study, 49 participants underwent radiofrequency microneedling device which delivered heat (40°–43°) for five different cycles for a total of two sessions 1 month apart. Patients rated the improvement as 74%, especially at the level of laxity and smoothness [75].

Another study comparing fractional radiofrequency microneedling with ER: YAG laser at the neck found higher patient and physician satisfaction with microneedling which yielded clinically better results [76].

### 4.2 Chest

Radiofrequency microneedling is also implemented for the rejuvenation of the décolletage. In one study, 12 subjects received a total of three sessions of fractional microneedling at least 3 weeks apart, using Endymed Intensif (EndyMed Ltd., Cesarea, Israel). The décolletage area was treated with 12–17 W during 110–140 ms. Overall improvement was noted in 67% of patients using the Global Aesthetic Improvement Scale (GAIS). Seventy percent of the participants also rated their treatment of skin laxity and rhytides as improved, and 60% of patients were satisfied with their skin texture [77]. To note, microneedling alone has not been used for cosmetic purposes for the chest in the literature.

### 4.3 Hands

There are little data in the literature about the use of microneedling on the hands. In one study conducted by Aust et al., three patients underwent microneedling for the hands. After the application of topical anesthesia, the rolling device was applied on the dorsum of the hand vertically, horizontally, and diagonally to produce thousands of micro‐wounds [78]. The total duration of the treatment is 20–30 min. Improvement at the level of texture and tightening of the skin was observed. However, further larger trials are needed to ascertain the effectiveness of microneedling and to point out its side effects at the level of the hands.

## 5 Chemical Peels

Skin peeling is a relatively easy, inexpensive, and fast method that has been used since the twentieth century to rejuvenate the skin [79]. Today, it is also a tool to treat various dermatological conditions such as acne scars, melasma, and hyperpigmentation [62]. Depending on their depth of penetration into the epidermis and dermis, chemical peels are classified into superficial, medium, and deep. Superficial peels work at the level of the epidermis. Examples include 10%–25% TCA, 40%–70% glycolic acid (derived from alpha hydroxy acid), or Jessner solution (composed of salicylic acid, resorcinol, and 85% lactic acid in 95% ethanol). Medium‐depth chemical agents penetrate the epidermis and the papillary dermis; examples are 30% TCA and 80% phenols. Deep chemical agents reach the mid‐reticular dermis and include 50% TCA and phenol–croton oil. Some chemical peels, such as TCA and salicylic acid peels, can leave a white “frost” on the skin, which is a sign of keratocoagulation (protein denaturation of collagen and keratin). Level of the frosting can be a visual indicator of the depth of penetration of the peel. Speckled white frost with mild erythema corresponds to a superficial depth. The goal of superficial chemical peels is to result in little to no skin frosting. When medium‐depth chemical peel, the frost becomes even with a background erythema while with deep chemical peel, the frost becomes solid with no background erythema [80]. The use of topical tretinoin improves the penetration of the acid and decreases the healing time, and also the wet soaks and emollients are recommended for epithelialization. Minor complications that may occur minutes after the procedure are erythema, irritation, and burning sensation [80]. Other complications to take into consideration include hypertrophic scarring, sun sensitivity, and infection [79]. Postinflammatory hyperpigmentation is a limiting factor to all chemical peels but especially to the medium and deep peels. For this reason, patients with Fitzpatrick IV to VI skin types are advised to undergo spot testing in a hidden area before treatment. After the procedure, it is important to avoid the sun, limit rubbing or scratching the skin, and apply a healing cream/ointment after the procedure at least twice daily. Absolute contraindications to chemical peeling include pregnancy and breastfeeding, history of keloids or hypertrophic scars, active infection, or immunosuppression (Table 5) [80]. The use of chemical peels on patients taking oral isotretinoin in the last 6 months remains a relative contraindication, as they can be used as a combination treatment for acne [85, 86].

**TABLE 5:** Chemical peel application techniques, adverse events, and post‐procedure care on the neck, chest, and hands.

Body part	Type of peeling	Acids involved	Application sites	Desired endpoint	Side effect	Follow‐up session
Chemical peeling						
Neck	Superficial	Phenol–croton oil 0.1–0.4 [81] TCA10‐30%	The upper and middle can be peeled stronger than the lower neck [82]	LIGHT chemical application [82]	In Fitzpatrick types 1 and 2, color mismatching between neck and face might happen [82]	Several sessions [82]
Chest	Superficial or light medium peels [81]	Glycolic acid with a concentration of 30%–70% is applied. followed by a neutralizing agent such as sodium bicarbonate or mixture of 70% glycolic acid with 40%TCA		Reach the papillary dermis. The thin top layer of the dermis (the inner layer of the skin). The papillary dermis has connective tissue and blood vessels that give nutrients to the epidermis (the outer layer of the skin) and that help control the temperature of the skin. Post‐peel consists of sun avoidance, and healing ointment at least twice a day [8]	Erythema as in another body part, pain, transient hyperpigmentation, and acneiform eruptions Infections are rare after superficial peeling [3]	
Hand	Superficial: Owing to the paucity of adnexal structures and thin epidermis of the hands [83]	35‐45%TCA Layered over 70% glycolic acid gel [83]		The desired endpoint occurred when erythema with scattered and speckles were observed Neutralization with 10% sodium bicarbonate solution [84]	No adverse effects were shown [83]	
		50% salicylic acid	Dorsal hands [28]			

### 5.1 Neck

The neck has an increased risk of scarring as compared to the face. For this reason, Barton et al. recommend the use of a light chemical application.

### 5.2 Chest

At the level of the chest, deep chemical agents are best avoided while superficial and medium‐depth peels are safe.

### 5.3 Hands

There exist few reports in the literature concerning chemical peeling of the hands [12]. In general, it is advised to avoid deep chemical peels on the hands. Swinehart et al. reported the use of 50% salicylic acid on the dorsal hands along with 48 h of occlusion in 11 patients. Erythema and maceration took up to 4 weeks to completely heal. However, they saw excellent results with a 90% decrease in photodamage and actinic keratosis. Because of the possibility of systemic absorption and subsequent salicylism, the risks and benefits of this procedure should be discussed with the patients [28]. Moreover, Sezer et al. compared 70% glycolic acid and 35% TCA chemical peel with cryosurgery for the treatment of solar lentigines of the hands. Three blinded investigators found no significant differences 2 months after treatment, but chemical peeling was preferred given its decreased risk of side effects [87]. Moreover, deeper peels confer the risk of scarring, dyschromia, the creation of a demarcation line between the hand and arm, and prolonged erythema [12].

## 6 Botox

Botulinum toxin is a purified form of the toxin produced by a spore‐forming gram‐positive bacteria, *C. botulinum*. In 2002, the FDA approved botulinum toxin for cosmetic use on facial wrinkles. Today, five types are available: OnabotulinumtoxinA (Botox), AbobotulinumtoxinA (Dysport), IncobotulinumtoxinA (Xeomin), RimabotulinumtoxinB (Myobloc), and botulinum toxin type A (Daxxify), which are widely used to rejuvenate non‐facial areas such as the neck and chest (Table 6). This non‐surgical lift with its little or no downtime, immediate results, financial, and time convenience is what makes it among the most common office‐based procedures. Adverse events include temporary edema and bruising, discomfort, and headaches [88]. Potentially rare dangerous adverse events include a systemic spread of the toxin leading to swallowing and breathing difficulties.

**TABLE 6:** Botox injection techniques, adverse events, and post‐procedure care on the neck and chest.

	FDA approved	Preparation	Volume needed	Injection sites	Method of injection	Follow‐up session	Side effects
Botox							
Neck	No	100 Allergan unit vial of Botox is diluted in a 2 mL volume or a 500‐unit vial of Dysport is diluted in 2.5 mL sterile saline [88]	3–10 units are needed for each injection and 50–100 units for the whole treatment	Four different injections are done 2 cm apart on one side of the mandibular border following a horizontal line	Patient is in sitting position then asked to clench his/her teeth to make the platysma bands more visible 1 mL syringe with a 30 G needle or microinjection technique in the entire area demarcated (Wu et al.)	In 15 days	Temporary edema and bruising, discomfort, and headaches [88] Rare cases of neck weakness, dysphagia, and dysphonia only in the case of deep injections (Ascher, Talarico et al.)
Chest	No	Onabotulinum toxin	7.5–10 U per injection with a total dose of 75–120 U per chest	Two injection lines: The first one is a horizontal line parallel to the intercostal line where the most caudal rhytide is visible The second line is V‐shaped starting presternally and ending at the intermammary level	The inferior part of the platysmal bands and the pectoralis major, which can be noticeable by asking the patient to cross their arms The muscle is tensed and injected tangentially using a 30 G needle		Temporary edema and bruising, discomfort, and headaches [88]

### 6.1 Neck

Brant et al. were the first ones to use Botox on the neck. Soon after in 2007, “The Nefertiti lift” by Levy emerged to complement the previously described technique. Using a 1 mL syringe with a 30 G needle, four different injections are done 2 cm apart on one side of the mandibular border following a horizontal line. The patient is then asked to clench his/her teeth to make the platysma bands more visible. The muscle is then held in between the index and thumb fingers and a series of vertical intradermal injections 2 cm apart is then performed. A blanching bleb then forms indicating that the correct depth has been reached. The approach to using BTX‐A (Botox, Allergan, Inc., Irvine, CA) involves injecting 3–10 U at each injection site with a 1/2‐in., 30‐gauge metal‐hubbed needle, and this is achieved by diluting 2.0 mL per 100‐U vial. An alternative method created by Wu et al. in 2015 can also be implemented which consists of microinjections, called “the microbotox” technique. This technique uses a 1:5 or 1:10 dilution of BTX‐A, where 100 U or 10 U are dissolved into a 5 mL solution, respectively [89]. In this technique, injections are not only done on the platysmal bands but in the entire area demarcated by the sternocleidomastoid muscle posteriorly, the depressor anguli oris medially, the upper border of the clavicle inferiorly, and a horizontal line drawn 5 cm above the mandibular border superiorly. Injections are done only 1.5 cm apart. A follow‐up after 15 days is appropriate. Rare cases of neck weakness, dysphagia, and dysphonia are reported; however, they are only the result of high doses of Botox or deep injections [90].

### 6.2 Chest

The major muscles in the chest are the inferior part of the platysmal bands and the pectoralis major, which can be noticeable by asking the patient to cross their arms [90]. The vertical and horizontal furrows of the chest are treated with botulinum toxin type A. Becker‐Wegerish et al. identify two injection lines onto which 15 U of onabotulinum toxin are injected, 2 cm apart: the first one is a horizontal line parallel to the intercostal line where the most caudal rhytide is visible. The second line is V‐shaped starting presternally and ending at the intermammary level. The muscle is tensed and injected tangentially using a 30 G needle [91]. In an international meeting done in 2010, a consensus was done for the injection. The panel of experts recommended a V‐shape injection consisting of 5–6 injection points per side, 7.5–10 U per injection with a total dose of 75–120 U per chest. They explained that the needle should be perpendicular to the muscle, injected 4 mm deep [90].

## 7 Profhilo (HA)

*Profhilo* is a type of injectable treatment launched in 2015 that contains high concentrations of hyaluronic acid. It is used for skin rejuvenation and helps to improve skin elasticity, firmness, and overall quality. *Profhilo* is unique in that it spreads through the skin, rather than just staying in one area, which allows it to treat multiple areas, of the face for example, with minimal injections. It is commonly used for anti‐aging purposes, particularly to treat fine lines, wrinkles, and sagging skin. *Profhilo* treatment typically involves two sessions spaced about 4 weeks apart, and the results can last up to 6 months. It is considered a safe and minimally invasive treatment option with minimal downtime (Table 7) [92, 93].

**TABLE 7:** Profhilo injection techniques, adverse events, and post‐procedure care on the neck.

Filler type	FDA approved	Preparation	Volume needed	Injection sites	Method of injection	Durability	Follow‐up session	Side effects
High‐ and low‐molecular‐weight hyaluronan intradermal injections (Profhilo)								
Neck	Yes	3.2% HA (32 mg H‐HA and 32 mg L‐HA in 2 mL of saline) [92]	2 mL (0.2 mL per injection site) [92]	10 injection sites on 3 vertical lines of the neck (3 laterally, 4 midline) [92]	Inject into the middle‐deep dermis using a 29 G needle and a bolus technique named Bio Aesthetic Point Neck [92]	6–12 months	Two sessions 1 month apart [92]	In the early stages, injection site reactions such as swelling, edema, redness, ecchymosis, and erythema may occur. Late‐onset local reactions such as swelling and nodules may also occur [93]

### 7.1 Neck

Sparavigna et al. investigated the efficacy and tolerability of hybrid complexes of high‐ and low‐molecular‐weight hyaluronan intradermal injections (*Profhilo*) for the treatment of skin roughness and laxity of the neck. The study involved 25 participants who received two injections 1 month apart. Results showed a statistically significant improvement in skin laxity, wrinkles, and hydration of the neck. Participants also reported high tolerability of the injections. The study suggests that hybrid complexes of high‐ and low‐molecular‐weight hyaluronan intradermal injections may be an effective and safe treatment option for improving skin texture and laxity in the neck area [92].

### 7.2 Chest and Hands

To note, no studies in the literature have been published for Profhilo body for use on the chest and hands.

Part 2 will continue with various types of lasers, providing specific details on parameters, follow‐up times, and some potential side effects.

## Conflicts of Interest

The authors declare no conflicts of interest.

## Data Availability

The data that support the findings of this study are available from the corresponding author upon reasonable request.
